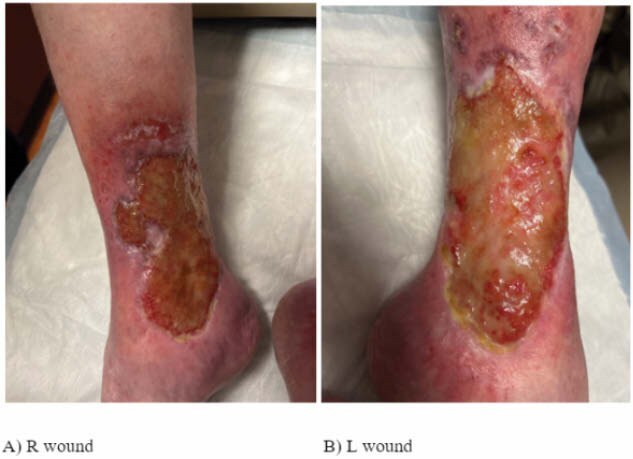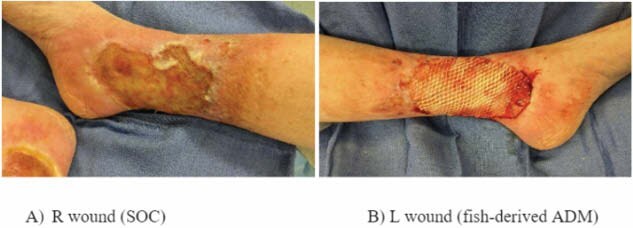# 835 Efficacy of Fish-Derived Acellular Dermal Matrix vs. Standard Care for Symmetric Venous Stasis Ulcers

**DOI:** 10.1093/jbcr/iraf019.366

**Published:** 2025-04-01

**Authors:** Anh-Tho Antoinette Nguyen, Derek Bell

**Affiliations:** Kessler Burn Center; Kessler Burn Center

## Abstract

**Introduction:**

Chronic venous stasis ulcers are complex and challenging to manage, often resulting in prolonged healing and poor outcomes with standard wound care. This case report presents a 66-year-old female with a medical history of pyoderma gangrenosum, hypothyroidism, mitral valve prolapse, ulcerative colitis, and chronic venous insufficiency, who developed symmetric venous stasis ulcers on both lower extremities. The patient had a long-standing ulcer on the left leg (15 years) and a newer ulcer on the right leg (4 years). After multiple unsuccessful treatment modalities, the patient was selected for a self-controlled study to compare the efficacy of a fish-derived acellular dermal matrix graft (fish skin graft) on the left leg versus standard wound care with hypochlorous acid and dry dressings on the right leg. The left leg ulcer underwent surgical excision and grafting with the fish-derived graft under general anesthesia, while the right leg continued receiving standard care.

**Methods:**

The left leg wound was excised and grafted with the fish-derived acellular dermal matrix. The right leg continued to receive standard care, consisting of hypochlorous acid and dry dressings. Weekly assessments included wound size reduction, percentage of epithelialization, wound bed characteristics, and patient-reported pain scores. Statistical analysis using paired t-tests compared the healing progress of the two treatments over a three-week period.

**Results:**

At three weeks, the fish skin graft-treated wound achieved complete closure and 100% epithelialization, while the wound under standard care showed only a 15% reduction in size with no significant epithelialization. Statistical analysis showed a significant difference in healing rates between the two treatments (p < 0.01). The weekly rate of wound size reduction was significantly higher for the fish skin graft-treated leg (mean 34.5%, SD ± 3.2%) compared to the standard care leg (mean 5.7%, SD ± 1.8%) (p < 0.001). Patient-reported pain scores decreased significantly for the grafted wound, with a mean reduction of 3.8 points on a visual analog scale (VAS) (p < 0.05), whereas no significant pain reduction was observed for the standard care wound (p > 0.05).

**Conclusions:**

The fish-derived acellular dermal matrix significantly accelerated wound healing compared to standard care, resulting in faster wound closure, reduced pain, and greater wound size reduction. This self-controlled case report underscores the potential of biologic grafts in improving outcomes for chronic venous stasis ulcers.

**Applicability of Research to Practice:**

This research highlights the potential of fish-derived acellular dermal matrices as an effective treatment for chronic venous stasis ulcers. By accelerating healing and reducing pain, this graft offers a promising alternative to standard care, encouraging clinicians to integrate advanced biologic treatments into routine wound management practices.

**Funding for the Study:**

N/A